# Loneliness and Social Isolation Detection Using Passive Sensing Techniques: Scoping Review

**DOI:** 10.2196/34638

**Published:** 2022-04-12

**Authors:** Malik Muhammad Qirtas, Evi Zafeiridi, Dirk Pesch, Eleanor Bantry White

**Affiliations:** 1 School of Computer Science & Information Technology University College Cork Cork Ireland

**Keywords:** passive sensing, loneliness, social isolation, smartphone, sensors, wearables, monitoring, scoping review, eHealth, mHealth, mobile phone

## Abstract

**Background:**

Loneliness and social isolation are associated with multiple health problems, including depression, functional impairment, and death. Mobile sensing using smartphones and wearable devices, such as fitness trackers or smartwatches, as well as ambient sensors, can be used to acquire data remotely on individuals and their daily routines and behaviors in real time. This has opened new possibilities for the early detection of health and social problems, including loneliness and social isolation.

**Objective:**

This scoping review aimed to identify and synthesize recent scientific studies that used passive sensing techniques, such as the use of in-home ambient sensors, smartphones, and wearable device sensors, to collect data on device users’ daily routines and behaviors to detect loneliness or social isolation. This review also aimed to examine various aspects of these studies, especially target populations, privacy, and validation issues.

**Methods:**

A scoping review was undertaken, following the PRISMA-ScR (Preferred Reporting Items for Systematic Reviews and Meta-Analyses extension for Scoping Reviews). Studies on the topic under investigation were identified through 6 databases (IEEE Xplore, Scopus, ACM, PubMed, Web of Science, and Embase). The identified studies were screened for the type of passive sensing detection methods for loneliness and social isolation, targeted population, reliability of the detection systems, challenges, and limitations of these detection systems.

**Results:**

After conducting the initial search, a total of 40,071 papers were identified. After screening for inclusion and exclusion criteria, 29 (0.07%) studies were included in this scoping review. Most studies (20/29, 69%) used smartphone and wearable technology to detect loneliness or social isolation, and 72% (21/29) of the studies used a validated reference standard to assess the accuracy of passively collected data for detecting loneliness or social isolation.

**Conclusions:**

Despite the growing use of passive sensing technologies for detecting loneliness and social isolation, some substantial gaps still remain in this domain. A population heterogeneity issue exists among several studies, indicating that different demographic characteristics, such as age and differences in participants’ behaviors, can affect loneliness and social isolation. In addition, despite extensive personal data collection, relatively few studies have addressed privacy and ethical issues. This review provides uncertain evidence regarding the use of passive sensing to detect loneliness and social isolation. Future research is needed using robust study designs, measures, and examinations of privacy and ethical concerns.

## Introduction

### Background

Humans are social creatures; thus, maintaining a healthy and positive social interaction is a necessary component of human existence [1]. Over the past few decades, loneliness has emerged as a global issue, and people rate it as a primary source of unhappiness in their lives [2]. Perlman defined loneliness as when an individual thinks that the quality or quantity of their social relationships is inadequate [3]. Loneliness or social isolation is a common problem that most people experience at some stage in their lives; however, it can be very distressing, especially when it becomes chronic [4]. In other words, loneliness is a distressing emotional state in which a person is dissatisfied with the proximity and pattern of their social interactions and relationships. Thus, loneliness is a fully subjective state of mind; a person can live completely alone and not experience loneliness, whereas another person may experience loneliness despite having a wide social network. Adult loneliness is a growing concern; according to a survey from the United Kingdom, 1 in every 20 adults reports feeling completely alone [5].

Social isolation refers to a person’s lack of social interaction with others at the individual or group level [6]. Social isolation can occur voluntarily or involuntarily and can have a positive or negative effect on an individual based on their mental and physical health [7]. Social isolation and loneliness are overlapping concepts often used interchangeably. Unlike social isolation, loneliness is always involuntary and involves negative emotions [8]. Although these two ideas are closely related, they do not have the same meaning. An individual can feel alone in a crowd, whereas another person can benefit from social isolation while experiencing solitude. Loneliness may occur when an individual is socially isolated for a prolonged period [9]. Loneliness is also a complex phenomenon that varies in intensity and is influenced by a variety of factors and conditions. To explain the multifaceted aspects of loneliness, Sadler and Weiss [10] distinguished between *emotional loneliness* and *social loneliness*. Emotional loneliness refers to the absence of close or intimate connections, whereas social loneliness refers to the absence of social networks. For instance, a child who has lost their mother experiences loneliness differently from a child who lacks playmates [11].

Loneliness is influenced by a variety of factors, including age, poor health, physical disability, relationship status, living alone, infertility, low wages, low levels of education, and socioeconomic status [12]. Certain personality traits, such as lower levels of extraversion and higher levels of neuroticism, may also increase the risk of loneliness [13]. According to a study on loneliness over the life span, it peaks in late adolescence, gradually declines during middle age, and then returns in later life [14]. As people age, they often lose social contact because of illness and cognitive decline, leading to loss of social relationships. Gradually, their social networks decline as they grow older, resulting in very few people from whom they receive support [15]. A report published by the Survey of Health, Ageing, and Retirement in Europe found that older adults living alone can experience higher levels of loneliness [16].

Loneliness has a detrimental effect on physical and mental health, increasing an individual’s risk of morbidity and mortality [17]. The effects of loneliness on cardiovascular health have been widely studied [18]. Poor social bonds have been linked to a 29% increase in incident coronary heart disease and a 32% increase in stroke according to a meta-analysis of longitudinal data on 35,925 people [19]. Loneliness can also result in various neuroendocrine problems and a weakened immune system [20]. In addition, loneliness can disrupt normal blood pressure, sleep patterns, and cortisol levels, resulting in serious health problems or even death [21]. Loneliness has been linked to various detrimental mental health outcomes [22], including depression [23], suicidality, less positive mood, poor sleep quality, poor overall physical health, and physiological abnormalities [13].

In addition to their primary function as communication devices, modern smartphones provide a plethora of capabilities [24]. A smartphone can be used as a handheld camera, a navigator, a fitness tracker, and a personal assistant [24]. As smartphones are equipped with a variety of powerful sensors, they have evolved into pervasive tracking systems [25]. Similarly, sensors in new wearable devices, such as smart watches and fitness trackers, have created the possibility of transforming them into a robust health tracking system [26]. Among the many fields that make use of the several capabilities of smartphones and wearables, one is passive sensing, the process of collecting data in the background using the ubiquitous nature of smartphones and wearables without the user’s active engagement. This concept of passive sensing or self-monitoring emerged from studies in the field of ubiquitous computing, where it has been called *context-aware computing* [27]. Smartphones can collect a vast amount of data regarding a user’s behavioral patterns, which can be modeled into bioindicators of the user’s well-being [28]. Using sensors, such as an accelerometer, a heart rate sensor, or a microphone as well as the GPS or Bluetooth connectivity as a proximity sensor, researchers collect high volumes of data about a user’s everyday life and behavioral patterns, such as social activities, time spent at certain places, daily health data, and a user’s activity log. All these capabilities of smartphones and wearables have made them a promising tool for tracking users’ health and well-being on a ubiquitous and passive level. In addition to smartphones and wearables, various environmental or ambient sensor-based devices have been used to passively gather information about users’ in-home behavioral habits, such as passive and active infrared sensors, pressure mats and tiles, and camera and microphone sensors, particularly for older adults [29].

In contrast to systematic reviews and other review-based approaches, scoping reviews are a relatively new tool for searching and summarizing the literature. Systematic reviews address specific questions using predefined methods to assess study quality. Scoping reviews can be used to map new or emerging fields of research and are useful when investigating a broader set of review questions that require the inclusion of studies from a wider range of study methodologies [30-32]. Researchers can conduct a scoping analysis in a recent or neglected research area for four primary reasons: to determine the design and purpose of the study, to determine the necessity of conducting a systematic review, to summarize the research results, and to locate research gaps within the existing literature [33]. This study investigated the broader field of loneliness and social isolation detection through passive sensing as a recent and less explored research area. This review aims to explore the types of passive sensing detection methods available in the literature for loneliness and social isolation, to investigate the target populations of these detection methods, to review how the developed systems were validated, and to find challenges and limitations of these detection systems.

### Loneliness Detection Approaches: An Overview

#### Assessing Loneliness in Research and Clinical Settings

There are several tools that assess loneliness. These assessments or scales are well established and have shown promising results in inferring loneliness. The University of California, Los Angeles (UCLA) loneliness measurement scale (version 3) is one of the most widely used in the general population and health care settings for assessing the frequency and severity of subjective loneliness in an individual [34-37]. Several other measures are available that may be used as a validated reference measure to determine an individual’s level of loneliness [38-41]. These scales include questions regarding marital status, social involvement in religious activities and clubs, social contact, social support, and social networks [28,42-46]. Most loneliness measures appear reliable. However, the evidence for validity is relatively limited, consisting mostly of comparisons between *at-risk* and normal populations or correlations with questions generating explicit self-identification as being lonely [47]. Moreover, we could not find any study that has used passive sensing methods to detect loneliness or social isolation in a larger, nonclinical population.

#### Technology-Based Detection Through Passive Sensing

Over the last decade, there has been increasing interest among researchers and clinicians in the opportunities presented by technology-based approaches for detecting loneliness and social isolation. Innovative technology-based approaches are extremely effective in terms of ubiquity and passive sensing [48] because they do not require the participant’s active participation. In the existing literature, most studies have used the following two methods to detect loneliness and social isolation: smart home–based methods (ambient sensors–based) and smartphone and wearable-based mobile sensing methods.

A smart home comprises a variety of in-home environmental or ambient sensors, along with more specialized audio, video, and biometric systems that can be used by family and caregivers to track older individuals’ actions and well-being while being physically away. A smart home can include a variety of sensors depending on the application. For instance, numerous studies have used in-home surveillance through video cameras [49,50], whereas others have used body-worn tags [51,52]. On the other hand, inexpensive ambient sensors allow a more unobtrusive method of monitoring behaviors in the home without user involvement. Ambient in-home sensors have been used for human behavior learning over the last few years, in which emotions, daily life patterns, or personality could be related to loneliness levels [53-55]. It incorporates an assessment of both physical and emotional well-being. These ambient in-home sensors are cost-effective, energy efficient, and easy to mount and maintain. They include motion sensors, which emit a signal whenever a motion is detected within the sensor’s coverage range; touch sensors, which generate a signal whenever a door is opened or closed; and pressure sensors, which emit a signal whenever a pressure threshold is crossed at the location of the sensor, which is usually in beds. Consequently, these sensors collect data on a user’s various behaviors, such as time spent in various areas of the home, sleep habits, time spent inside and outside the home, and in-home mobility patterns. These activity patterns can ultimately serve as measures or biomarkers of loneliness or social isolation.

Researchers have also used mobile and wearable devices for health monitoring over the last few years. The rapid proliferation of smartphones and wearables, such as fitness trackers, which are equipped with powerful sensors, may provide another pathway for detecting loneliness and social isolation. The data passively acquired from mobile sensors can be modeled into various behavioral habits that can be used to identify lonely individuals. Behavioral patterns included a participant’s social experiences, mobility patterns, and frequently visited places.

## Methods

### Search Strategy and Data Sources

The protocol for this scoping review was developed as per the guidelines of the PRISMA (Preferred Reporting Items for Systematic Reviews and Meta-Analyses) [56] and PRISMA-ScR (Preferred Reporting Items for Systematic Reviews and Meta-Analyses extension for Scoping Reviews) [57]. The databases IEEE Xplore, Scopus, ACM, PubMed, Web of Science, and Embase were searched. Articles published between January 2011 and December 2021 were extracted independently by the authors MMQ and EZ. The search terms and results for each database are presented in Table 1. The search criteria were based on loneliness, social isolation, and detection-related keywords.

**Table 1:** Search keywords and result statistics for computer science and social science databases.

Database and keywords combination			Search results, n
**IEEE Xplore**			
	*Loneliness AND Detection*	13	
	*Loneliness AND Sensing*	27	
	*Loneliness AND Passive Sensing*	1	
	*Loneliness AND Monitoring*	15	
	*Social Isolation AND Detection*	56	
	*Social Isolation AND Sensing*	41	
	*Social Isolation AND Passive Sensing*	1	
	*Social Isolation AND Monitoring*	65	
**Scopus**			
	*Loneliness AND Detection*	4373	
	*Loneliness AND Sensing*	725	
	*Loneliness AND Passive Sensing*	61	
	*Loneliness AND Monitoring*	6443	
	*Social Isolation AND Detection*	5501	
	*Social Isolation AND Sensing*	834	
	*Social Isolation AND Passive Sensing*	51	
	*Social Isolation AND Monitoring*	5901	
**ACM**			
	*Loneliness AND Detection*	703	
	*Loneliness AND Sensing*	1339	
	*Loneliness AND Passive Sensing*	27	
	*Loneliness AND Monitoring*	603	
	*Social Isolation AND Detection*	313	
	*Social Isolation AND Sensing*	631	
	*Social Isolation AND Passive Sensing*	10	
	*Social Isolation AND Monitoring*	357	
**PubMed**			
	*Loneliness AND Detection*	220	
	*Loneliness AND Sensing*	770	
	*Loneliness AND Passive Sensing*	8	
	*Loneliness AND Monitoring*	200	
	*Social Isolation AND Detection*	2238	
	*Social Isolation AND Sensing*	1981	
	*Social Isolation AND Passive Sensing*	24	
	*Social Isolation AND Monitoring*	1202	
**Web of Science**			
	*Loneliness AND Detection*	109	
	*Loneliness AND Sensing*	1130	
	*Loneliness AND Passive Sensing*	16	
	*Loneliness AND Monitoring*	299	
	*Social Isolation AND Detection*	350	
	*Social Isolation AND Sensing*	1195	
	*Social Isolation AND Passive Sensing*	13	
	*Social Isolation AND Monitoring*	701	
**Embase**			
	*Loneliness AND Detection*	137	
	*Loneliness AND Sensing*	22	
	*Loneliness AND Passive Sensing*	1	
	*Loneliness AND Monitoring*	235	
	*Social Isolation AND Detection*	390	
	*Social Isolation AND Sensing*	34	
	*Social Isolation AND Passive Sensing*	1	
	*Social Isolation AND Monitoring*	704	

### Inclusion and Exclusion Criteria

Studies were included if they were published in the English language and presented results on identifying loneliness and social isolation using passive sensing technology, such as smartphone apps, fitness trackers, and home sensors. Studies were excluded if they were written in a non-English language, if loneliness or social isolation was not one of the assessed outcomes, or if the detection method was other than passive sensing.

### Data Extraction

**Figure 1:**
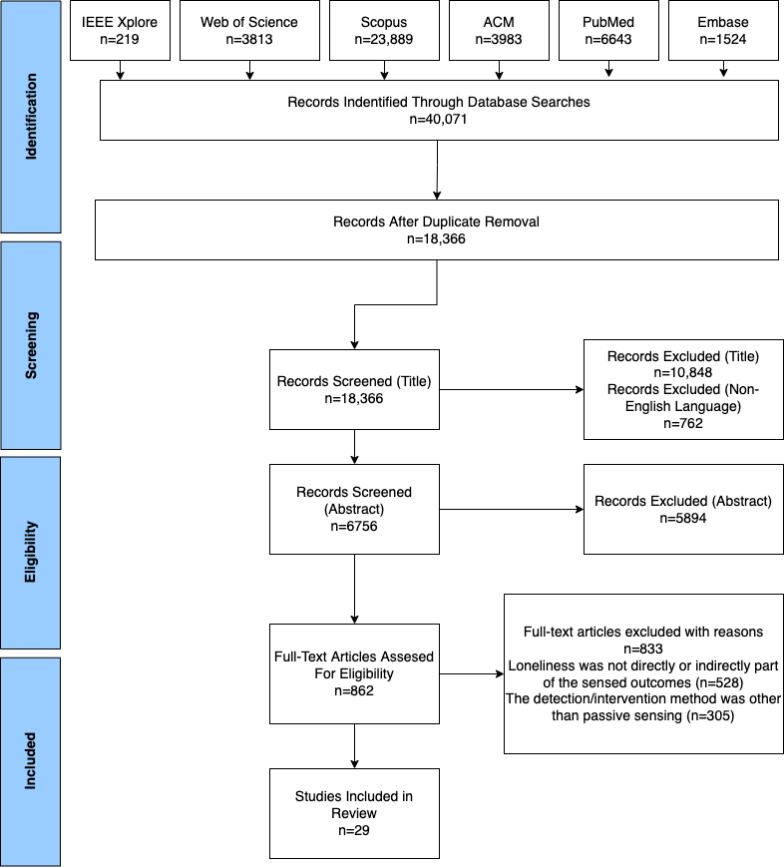
Flow diagram of the literature search and selection process (adapted from PRISMA [Preferred Reporting Items for Systematic Reviews and Meta-Analyses]).

The selection process is illustrated in Figure 1. A total of 40,071 studies were identified using the search terms. After title and abstract screening, 862 full texts were reviewed, of which 29 (3.4%) studies were selected for inclusion in the review, as follows:

- General description of the study: authors, year, and country
- Study design: population type and age, participant sample selection process, duration of the study, ground truth data collection methods, and privacy handling
- Study technology insights: technology used for sensing, data collection streams, and algorithm.
- Study outcome characteristics: indicators or identification markers and sensed outcomes.

## Results

Multimedia Appendix 1 [58-85] summarizes the main information from each study to address the questions under investigation.

### Population

Most studies targeted young adults (10/29, 34%), most frequently college students [58-67], or older adults (12/29, 41%) [68-78,86], whereas 17% (5/29) of the studies included a mixed age group [79-84]. The studies sampled between 5 and 364 participants and lasted anywhere from 1 week to 5 years. In all, 3% (1/29) of studies was not longitudinal [61], and 7% (2/29) of other studies did not report the duration [75,81].

In all, 37% (11/29) of studies explicitly stated the participants’ ages [61,62,64,67,68,70,75,76,78,83,84], whereas the other studies (18/29, 62%) mentioned only the age group without providing their age: younger adults [58-60,63,65,66], older adults [69,71-74,77,86], or mixed age groups [79-82]. Of the 11 studies, a total of 4 (36%) of studies focusing on a younger population reported an age range of 18 to 28 years [60-62,64], whereas 6 (55%) studies with a target sample of older adults reported an age range of 53 to 91 years [68,70,75,76,78,83]. Participants in 9% (1/11) of studies ranged in age from 18 to 78 years, representing a mixed group of younger and older adults [84].

With respect to the participants’ gender, a total of 3 studies focusing on a younger population included both male and female participants [60,63,83]. Female participants dominated 3 studies [59,61,67], male participants dominated 2 others [64,66], and 1 study had exclusively female participants [62]. Female dominance has been clearly observed in older population studies. A total of 7 studies concerning older adults reported their gender, and all (7/29, 24%) these studies showed a female predominance, except one. The female ratios in these studies were 60% [70], 81% [72], 74% [68], 54% [74], 89% [75], 44% [78], and 88% [76].

A total of 11 studies mentioned participants’ educational level [59,63-68,72,76,78,83]. The younger population mostly comprised college students [59,63-67]. In all, 2 studies recruited first-year college students [59,63], whereas 4 others targeting a younger population recruited a mix of university students at the graduate and undergraduate levels [61,65-67]. Only 4 studies reported the education levels of the older adults [68,72,76,78], of which 2 (50%) studies included participants who had completed their graduate studies [72,78], and the remaining 2 (50%) studies included participants with varying levels of education, with 46% (12/26) completing a college education in a study [76], 39% (19/50) completing primary level education, and 39% (19/50) having had no formal education in another study [68].

Participants’ ethnic status varied. A total of 8 studies clearly reported the origin of their participants [59,60,63,65,66,68,75,78], 5 (63%) of which targeted a younger population from diverse ethnic groups [59,60,63,65,66], and 3 (37%) of which targeted older adults [68,75,78]. In the younger population, most participants had an Asian cultural background, whereas 2 studies were from the United States with participants from different backgrounds [59,66], 1 study was from China [65], and 1 was from Singapore [63]. The remainder were White, Hispanic, African American, or Caucasian [60,65]. A study from Singapore examining an older population had 87% Chinese participants [68], whereas another US-based study included 75% White participants [75].

### Sensing Streams

Given that smartphones and wearable fitness trackers are equipped with a variety of physical sensors, they are highly effective instruments for monitoring users’ movements and passively collecting data on their daily life patterns. Of the 29 studies, 18 (62%) used smartphones or wearables as a sensing modality to passively capture data [58-67,71,74,79,80,82-84,87].

Among the various physical sensors used in smartphones or wearable devices, the accelerometer and GPS are the most frequently used sensors for passive data collection. Indeed, 72% (13/18) of the studies that used a smartphone or smart watch as a sensing instrument made use of accelerometer and GPS sensors to gather data on a user’s physical activities [59-63,65-67,74,80-83]. The accelerometer is used because of its low power use and low privacy concerns, whereas GPS is used to monitor the users’ regularly visited locations and the time spent at those locations, which aids in determining their social habits. Another explanation for using GPS in several studies is that it is typically available with all smartphones.

Following this, Bluetooth and microphones are the second most widely used sensors in the identified studies, accounting for 39% (7/18) of studies that used passive sensing via smartphones or wearable devices to acquire user data [59,62,65-67,80]. Bluetooth is typically used as a form of proximity sensor to gather information about a user’s sociability and physical encounters with other users who also have Bluetooth enabled in their devices, whereas a microphone is used to infer sleep and social experiences. In addition, 7% (2/29) of studies used Wi-Fi MAC addresses to collect information about users’ sociability. Moreover, a study collected data from a smartphone light sensor to detect the screen lock or unlock status [82].

Apart from the sensors in smartphones, the studies included data on phone activity, which could also be used as an indicator of loneliness or social isolation. SMS text messages and call logs are the most frequently used; 56% (10/18) of the studies have used these logs to derive user communication activities [59,60,62-65,71,74,76,82]. In addition, 2 experiments integrated the number of times that each smartphone was locked and unlocked [59,82], and 2 studies gathered data on the types of apps that were used more often to ascertain users’ mood and sociability levels [65,82]. To maximize learning opportunities regarding user habits, a study collected contact information, browsing history, and email-related data [64].

Of the 29 studies included here, 9 (31%) solely used ambient and other physical sensors installed at different locations in the home to learn about participants’ in-home mobility patterns and behavior [68-70,72,73,75,77,81,86]. Different types of sensors were used, including passive infrared motion sensors installed on walls to collect motion data, pressure sensors to detect the presence of the participant on a bed or chair, sound sensors to detect social interactions and activities in the household, and door sensors to detect walking patterns and people movements.

### Loneliness and Social Isolation Assessment

Different assessment methods have been used in experiments to validate the passively collected data. Most studies (21/29, 72%) relied on self-reports and questionnaires, which can be administered directly in a clinical setting or remotely using a mobile app. Self-reports or questionnaires were used to gather data on participants’ physical and mental health in some of the included studies (8/29, 27%) that assessed social interactions and well-being [60,67,68,71,80,82-84], depression or anxiety [60,62,65,66,70,77,78], and daily activities [63,67,68,79,80,82]. Numerous scales have been used specifically for the detection of loneliness, including the UCLA loneliness scale [88], the De Jong Gierveld Loneliness Scale [41], and the ESTE-R Loneliness Scale [74]. The Social Interaction Anxiety Scale [89], Depression Anxiety Stress Scale [90], and Positive Affect Negative Affect Schedule [91] have been used to measure depression and anxiety symptoms in individuals. Some studies used self-developed questionnaires, such as the ecological momentary assessment, which were specifically related to the application domain and target populations.

### Efficacy and Reliability

#### Detection Methods

Numerous markers were used in the included studies to identify changes in behavior, which may infer loneliness or social isolation. Of the various markers, the number of incoming and outgoing calls and SMS text messages is the most widely studied, with 24% (7/29) of studies () using this metric for detecting loneliness [60,63,64,72,74,76,82]. Similarly, time spent outside the home is also the most explored marker; 24% (7/29) of studies presented the relationship between time spent outside the home and its effect on loneliness levels in individuals [68,72-75,83,86]. Additional markers include sleep cycles that have been examined in 4 studies [59,66,68,70], voice activity and verbal communication explored by 4 studies [62,63,66,83], activity, mobility, and walking speed investigated in 6 studies [59,61,62,70,72,80], the average time spent in different areas of the household [68,71], and app types that have been studied by 2 studies [65,82]. Interaction with others detected through Bluetooth proximity sensing was examined in 5 studies [59,62,63,65,67], and 3 studies investigated the effect of speech activity on loneliness [62,63,83].

#### Phone Calls or SMS Text Messages

A total of 8 studies examined the relationship between incoming and outgoing phone calls and loneliness [64,65,71,72,74,76,82,84]. According to a study, individuals who received fewer incoming calls rated themselves as being more lonely on loneliness measurement scales compared with those who received more calls [82]. Another study found that loneliness was associated with fewer incoming calls but not with increased outgoing calls. The most lonely individual received only 40% of the number of calls received by the least lonely individual [76]. Another study explored the relationship between loneliness and calls from family members and friends. Outgoing and incoming family calls are significant characteristics of family and spousal loneliness, which is about feeling lonely within intimate relations. Similarly, for social loneliness, calls from friends were not considered a relevant attribute; rather, calls from acquaintances were considered a relevant attribute while developing predictive models for loneliness detection [74]. A total of 8 studies investigated the relationships between call frequency and call duration with loneliness. Individuals with greater loneliness received fewer calls on average according to a study [76]. There is a weak negative correlation between loneliness and call length, indicating that people who make longer calls have lower feelings of loneliness. In comparison, loneliness showed no significant relationship with the frequency of phone calls [65]. Another study found a similar negative correlation between phone conversation duration and social well-being [84]. In addition, 2 other studies found a similar negative correlation between the length of calls and loneliness on the UCLA loneliness scale [64,71]. Similarly, the frequency of phone calls was significantly linked to the length of time spent on the phone [72]. Another study found that students who received more phone calls, particularly on weekdays, reported reduced feelings of loneliness, better transition to college life, and a stronger sense of class community [63]. Regarding SMS text messages, individuals with a high score for social anxiety or loneliness rarely receive messages [82]. Another study discovered a weak negative correlation between loneliness and SMS text messages, particularly at night [65]. Although another study found no significant relationship between instant messenger and SMS text message frequency and loneliness or social well-being, phone call use was shown to be favorably associated with social well-being [84]. Another study discovered a significant correlation between the quantity of messages and UCLA loneliness scale [64].

#### Web Social Activity and Communication Apps

A total of 3 studies examined the relationship between web social engagement and loneliness [65,82,84]. According to a study conducted with a younger population, loneliness is positively linked to app use frequency, which implies that younger individuals who spend more time on social apps experience more loneliness [65]. However, a study targeting older adults found the opposite: older adults who spent more time on social media had substantially reduced feelings of loneliness [84]. The authors found that older adults’ social media networks led to reduced feelings of loneliness and increased levels of general well-being compared with younger adults’ social media networks [84]. Another study found that smartphone behaviors indicative of web social activity (eg, the frequency of using social or communication apps) were unrelated to social anxiety or loneliness [82].

#### Number of Computer Sessions or Hours on the Computer

In addition, 2 studies evaluated computer use in terms of the number of sessions and overall time spent on the computer daily [72,78]. They discovered that an increasing number of computer sessions was associated with higher levels of loneliness. Previous research on the relationship between computer use and loneliness has produced contradictory results. Although some argue that computer use helps combat loneliness [92], others argue that increasing computer use (particularly among young people) is associated with increased loneliness [93-96]. According to a study, telephone use and computer use were not shown to be substantially related to loneliness [72]. The authors stated that the lack of significance for these social factors may be explained in part by the strong correlation between them. That is, the number of phone calls was significantly associated with the amount of time spent on the phone, whereas the number of computer sessions was significantly associated with the amount of time spent on the computer [72].

#### Bluetooth Proximity Sensing

A total of 4 studies examined the association between Bluetooth proximity-sensing social interactions and feelings of loneliness [58,59,65,67]. According to a study, when a number of different Bluetooth devices are identified in the vicinity, people tend to report feelings of loneliness. This tendency may represent situations in which a person feels lonely in public places with a high concentration of Bluetooth devices [67]. Another study discovered a significant association between Bluetooth device encounters and the Patient Health Questionnaire-9 depression scale [58]. However, a study found no relationship between Bluetooth proximity and loneliness [65]. A study used Bluetooth together with other sensor data, such as GPS, Wi-Fi, SMS text messages, and call logs, which showed better accuracy in predicting loneliness levels in individuals [59].

#### Daily Phone Use or Time on Mobile Phone

A total of 3 studies have explored the relationship between general smartphone use and loneliness [59,65,76]. According to a study, loneliness in older adults is associated with reduced daily phone use, to the point where the most lonely individual uses the phone almost two-thirds less than the least lonely one [76]. Another study found that decreased phone use during certain weekends and morning hours was associated with increased loneliness among the younger population [59]. Similarly, another study on students discovered that those at risk of loneliness spend more time on their smartphone each day and that loneliness was positively associated with app use, irrespective of the time of day [65].

#### Demographic Characteristics Affecting Loneliness

Of the 29 studies, 4 (14%) assessed gender differences in loneliness [76,82,84,87]. According to Petersen et al [76], gender had a substantial effect on the daily number of calls, with women making or receiving twice as many calls as men. Another study compared the gender of participants in high- and low-loneliness groups; the result of the chi-square test on the gender-based difference revealed no significance [82]. Another study found that women with a greater sense of well-being use reading apps and browsers more often than men do [87]. Similarly, 4 studies examined passive sensing and participants’ behaviors in terms of age. A study assessed the ages of participants in high- and low-loneliness groups; the chi-square test indicated no statistical significance for the age difference [82]. According to another study, age was not a significant predictor of the daily number of calls [76]. In a study by Wetzel et al [84], the authors discovered a substantial relationship between participants’ age and social media use time, suggesting that the association between social media use and perceived loneliness varies by age. This indicates that older adults who spend more time on social media experience substantially reduced feelings of loneliness. At younger ages, more time spent on social media was associated with increased levels of perceived loneliness. Another study found that the activity and frequency of messages and calls increased with the age of participants born before the year 2000 [79]. Cognitive ability was included as a demographic variable in 2 studies. According to a study, people with a chronic illness report significantly higher feelings of loneliness and poorer levels of social well-being than those without a chronic condition [84]. Another study discovered a relationship between better cognitive performance and higher daily call frequency [76]. In addition, this research examined whether an individual’s pain level might serve as a predictor of phone calls but discovered no significant relationship between pain level and call frequency [76]. A total of 2 studies examined the relationship between an individual’s personality traits and activity patterns inferred from passively collected smartphone data [64,79]. Moreover, a study discovered a significant correlation between emotional stability and extroverted personality characteristics and most smartphone-sensed features. In comparison, agreeableness, conscientiousness, and intellect personality traits are slightly associated with most of the features sensed by the smartphone, such as the number of messages, number of browser searches, number of calls, number of long incoming or outgoing calls, and number of contacts [64]. According to another study, gratitude is associated with participants’ message and call patterns, with the most grateful participants communicating mostly through their smartphones [79]. Regarding occupational status, participants who are retired, are homemakers, or are unemployed are typically the least active and more prone to loneliness, whereas those enrolled in college or working full-time or self-employed are typically the most active with lower levels of loneliness. This analysis was performed using smartphone sensor data collected from an accelerometer, GPS, microphone, SMS text messages, and call logs [79]. Another study that used smartphone app communication data as an indicator of loneliness across the adult life span during the COVID-19 pandemic found that individuals who did not have a partner reported greater feelings of loneliness and poorer social well-being than those who had a partner [84]. The reason for this might be that during the COVID-19 pandemic, everyone was required to stay at home, and those without a partner were more prone to experiencing loneliness than those in a relationship.

#### Time Outside of Home and Time Spent at Home

A total of 7 studies examined the effect of time spent outdoors or at home on an individual’s loneliness levels [68,72,73,75,77,83,86]. A study with older adults found that spending more time away from home was associated with decreased levels of loneliness [75]. Similarly, other research with both young and older participants discovered that people who spent more time outside the home reported fewer feelings of loneliness [83]. Other research has analyzed older adults’ outdoor trips using two measures: average daily outdoor time and number of outdoor visits. They discovered that spending more time outdoors resulted in decreased levels of loneliness and higher social networking scores. In addition, 2 other studies discovered a strong association between the daily time spent outside the house and loneliness [72,77]. Similarly, another study discovered that individuals who spend more time at home are lonely. The physical activity score is positively associated with the average amount of time spent outside the house, suggesting that outings are also an important indicator of physical activity [86]. A preliminary analysis found no relationship between the time spent outside the home and loneliness [73]. They stated that their participants were more technology literate and may have engaged in social activities such as computer or mobile use that do not require leaving the home.

#### Daily Activity and Movement

A total of 5 studies investigated the effects of daily activities and movements and their duration on loneliness levels [59,65,66,68,83]. A study discovered that individuals performing more physical activities experience less loneliness [66]. Similarly, greater dispositional loneliness (UCLA loneliness scale) was associated with a substantially shorter mean movement duration in another study [83]; dispositional loneliness is defined by a sense of disconnection from others and distressing emotions of isolation [97]. In addition, loneliness increased with the number of significant places visited [83]. Loneliness is also negatively correlated with movement duration [83]. Other research conducted with students discovered a strong negative correlation between loneliness and indoor mobility; for example, the amount of time a student moves indoors throughout the day. Research indicates that inactive students are more likely to experience loneliness. Another study found that people who spend more time engaging in activities are less lonely regardless of the time of day or night [65]. Another study found a similar pattern: a higher total amount of movement and steps throughout the day and night time hours results in less feelings of loneliness [59]. Deviation from one’s regular geospatial activity was associated with a substantial reduction in daily stress and loneliness [68].

#### Conversation Activity

A total of 3 studies investigated the effect of conversation and its duration on loneliness levels [58,66,83]. In addition, 2 studies found no significant correlation between speech duration and loneliness [66,83]. However, another study found that students who engage in less conversational contacts are more likely to experience depression [58].

#### Sleep

A total of 3 studies investigated the relationship between sleep duration and feelings of loneliness and stress [58,66,68]. According to a study, sleep duration was not correlated with loneliness but was negatively related to daily stress, meaning that people who get enough sleep experience less stress [66]. Another study with students discovered that those who sleep fewer hours are more prone to depression [58]. A study focusing on older adults found that those who perceived themselves as socially isolated had more midday naps [68].

#### Privacy Issues and Ethical Concerns

Overall, 69% (20/29) of the studies did not address privacy or ethical issues and did not explicitly mention privacy concerns [59,64-69,71-83]. A total of 5 studies provided details of ethical approval from the relevant committees and participant consent [60-62,70,84]. In addition, 2 studies stated that they maintained user data on a secure data server and anonymized user identifiers to protect users’ privacy [58,85]. Moreover, a study collected statistical data on smartphone use only to preserve participants’ privacy [63]. Another study, which uses a video camera placed in a smart home to monitor users’ activities, reported that they captured footage for only 5 seconds whenever a motion was detected at the door [86].

## Discussion

### Comparison With Previous Work

Numerous review studies have examined the use of passive sensing to monitor a variety of mental and physical health and well-being outcomes. Many aspects of passive sensing have been covered in previous review studies, providing researchers with current challenges and suggestions for potential future research. These reviews have focused on different mental and physical health conditions, such as stress [98], mood disorders [99], sleep problems [100], cardiac issues [101], chronic health conditions in older adults [102] and schizophrenia [103]. Compared with previous reviews, this review is the first to focus on the use of passive sensing for loneliness and social isolation detection and to explore the limitations of the passive sensing systems used.

### Findings From Reviewed Studies

The targeted population is one of the key dimensions to be considered when designing and developing loneliness detection systems. Most studies (17/29, 58%) included in this scoping review targeted younger populations. It may be relatively easier to generate a sample group of younger people from colleges or universities, as opposed to older adults, who are dispersed across community settings and may not be readily accessible through services, education, or workspaces. Evidence in the literature suggests that isolation is typically higher in late adolescence and after retirement than in the middle age range [14]. Moreover, unemployed individuals in the middle age group are more susceptible to loneliness [104]. An age-related association was observed in the detection methods; loneliness in younger adults was assessed mostly through smartphones [58-60,62,63,65-67], whereas ambient sensors were used more frequently by older adults [68-78,86]. Ambient sensing may be used by older people because they are less likely to use wearables and have higher privacy concerns [105]. Some older adults may not be able to use smartphones because of lack of technological literacy and complex user interfaces [106]. In addition, people with dementia can forget to carry or wear such devices or charge their smartphones in a timely manner, resulting in intermittent or no data collection. Furthermore, there is a strong possibility that some wearable sensors create an unpleasant feeling during prolonged skin connection (eg, electrodes on the skin). As a result, older adults may reject such wearables, particularly at home [107].

Before they are widely available to the public, these loneliness detection systems must undergo extensive validation to ensure that users feel comfortable using and trusting them. The validation of such detection systems is important because it provides relevant input and knowledge about the performance and reliability of the systems to researchers and developers. Two facets of validation of these loneliness detection systems should be discussed: participant selection and technology’s efficacy in assessing social outcomes. With regard to population selection, most of the included studies (17/29, 58%) recruited younger adults and college students via advertisements on student mailing lists or Facebook groups. Moreover, most of the studies recruited first-year college students or undergraduate students from the same college or university, with a random ratio of male and female participants. A study recruited students from a specific class only [58], resulting in a population with very similar sociodemographic backgrounds, daily routines, and study challenges. There is a lack of detail on randomization in student population selection, either in terms of sample collection from more than one college or from younger populations other than college students. A study recruited international students because they were considered at a higher risk of loneliness [64]. In addition, many studies (9/29, 31%) did not include sufficient information on the participants’ socioeconomic status. In the case of older adults, studies recruited participants who resided in sensor-enabled homes created by specific senior citizen welfare organizations. Some studies (6/29, 20%) omitted older adults with physical, sensory, or cognitive impairment, which may have resulted in demographic selection bias because people with disabilities may experience greater loneliness [108,109]. Most studies (9/12, 75%) have focused on older adults who were already living alone to assess their loneliness, which may result in biased findings. Few studies (3/29, 10%) used the Mini-Mental State Examination [110] to screen and exclude older adults with cognitive impairment. In addition, some studies (7/29, 24%) have not mentioned their sample selection or recruitment process for both the younger and older populations; instead, they simply reported the number of individuals from a specific age group.

Most studies (24/29, 82%) have used supervised machine learning techniques to infer loneliness levels from passively acquired data. In supervised machine learning, labeled data are required to train a model, where the gathered ground truth data are used to label the raw sensor data. Ground truth data have been used to compare and validate passively acquired data through smartphones and other passive devices. Inaccuracies in the ground truth evidence result in incorrect model training and an inefficient detection system. Thus, ground truth data were critical in these studies. Ground truth data from assessments, such as loneliness assessment questionnaires, are used to label the data collected through passive sensing streams with the corresponding ground truth state. Most studies (21/29, 72%) have used self-reports and questionnaires, which can then be used for validation purposes with passively collected data. This method has some drawbacks, such as users may not always respond to such questionnaires, or there could be accuracy issues depending on the participants’ current mental and physical state.

Participants’ motives, preferences, and expectations regarding surveillance systems can influence their willingness to use available solutions. Some of those interests could be physiological, such as improving behaviors in daily life, and some could be more technical, such as system scalability and acceptability. Most studies (18/29, 62%) that use smartphone apps to passively acquire user data have used Android-based mobile apps because of their lower privacy restrictions compared with the iPhone iOS operating system when collecting data through various sensors available on smartphones. Another reason might be cost, as Android devices are more affordable and are the most widely used operating system, encouraging more users to use the proposed systems.

Another technological consideration of smartphone-based detection systems is their power use. Owing to continuous data collection and constant use of energy by various sensors, the battery life of smartphones degrades more quickly, which is a major concern for users. Only a few studies (6/29, 20%) have addressed this topic or plan to do so in the future. The approach used to reduce power use is to reduce the sampling rate of sensors when the battery level falls below a certain threshold or to use less beneficial sensors only once a day [48].

Apart from energy use, the participants’ primary concern is privacy [48]. Although most of the reports did not mention privacy concerns, few studies (8/29, 28%) indicated that this is one of the key concerns of participants. These studies indicated that they obtained approval from participants for passive data collection or that they obtained privacy and ethical clearance from an organization. Several (6/8, 75%) of these studies used anonymous participant identifiers to store data and transmitted them securely to the server. However, a sizable portion of the studies did not address privacy concerns, which raises questions about their acceptability to participants and the wider public and about the potential harms for this detection method in the case of data and privacy breaches.

### Implications for Future Research

This review demonstrates that previous research has not drawn strong distinctions between social isolation and loneliness, with many researchers using both terms interchangeably. According to previous research, loneliness and isolation overlap relatively little, with most lonely times occurring when not alone, for example, when an individual is in a crowd of strangers [67]. Across all self-reports, loneliness seems to be greatest when participants also report being alone at the time and lowest when participants additionally report being with a significant other (partner or friend) [67]. In addition, some experiments have intertwined loneliness with other related concepts. For instance, in a study [111], the authors recorded the number of visits to older adults living alone and did not attempt to explicitly detect loneliness. Thus, it is necessary to distinguish loneliness from other similar terms, most notably, social isolation.

Another significant gap pertains to privacy concerns. Most research has not focused on privacy issues. A few studies (9/29, 31%) discussed their strategies for maintaining device and data privacy, which included anonymization and safe data transmission to data collection servers; however, most of the studies (18/29, 62%) plainly stated that they gathered data with the consent of the users but did not discuss data privacy and security. Most studies (12/18, 66%) collecting participant data passively through smartphones and wearables were based in the United States, China, and Asia, with very few studies conducted in European countries. This may be because the European Union’s General Data Protection Regulations place stringent restrictions on data collection and transfer. It is important to engage potential study participants in the design, development, and validation phases of such systems to ensure that the system satisfies their expectations [112]. This is crucial if the system is to be adopted extensively and sustainably by the target population.

Many smartphone or wearable users prefer tracking systems to provide them with valuable knowledge and feedback about their activities and well-being status [113]. When people receive well-being-based feedback, they are more likely to make positive changes in their lifestyle and behaviors related to physical activity, including well-being, sociability, and mental health [114,115]. However, little is known about the impact of user feedback on behavioral changes related to loneliness. As demonstrated in this scoping review, existing systems are deficient in delivering real-time feedback to participants, which can help participants develop interest and trust in such tracking systems and help them to make attempts to change their lifestyle in response to system recommendations. Experts are examining ways to develop these tracking systems to provide valuable feedback, alerts, and advice to users to improve their current mental health [116].

Most of the studies (25/29, 86%) in this scoping review were pilot or feasibility studies with very small or limited sample sizes. The validation of these detection systems is a crucial component, and the systems should be validated with a subset of the target population that is highly representative over an adequate period to obtain appropriate data to deliver as reliable results as possible. Many studies are of short duration, which may provide an insufficient window for detection and build participants’ trust and acceptability in detection systems. In addition, some of the proposed systems selected population samples from a very specific population type or at a specific period, such as a lockdown during a pandemic crisis [84], which may result in misleading outcomes when applied to other populations or at different periods. Similarly, the population selection methods used were not rigorous, resulting in potential self-selection bias and limited generalizability, particularly with low representations of at-risk groups, such as unemployed people or older adults not in supported housing.

### Limitations

Although we conducted a thorough search of computer science, health, and social science databases using multiple search terms related to loneliness and social isolation detection, some articles could have been overlooked. For example, studies that focused on general mental health or emotion recognition, with loneliness or social isolation serving as a subset of larger research, were not included. Furthermore, because we included only studies conducted in English, there might be several studies on loneliness detection published in other languages that were not included in this scoping analysis.

### Conclusions

It is evident that the use of smartphones, wearable smart devices, and ambient sensors to detect loneliness and social isolation in different age groups has increased in the last few years. Compared with more conventional tracking systems, smartphones are simple to use, unobtrusive, familiar, and inexpensive. They also have a variety of sensors that allow the collection of users’ data in real time, without interfering with users’ daily activities. This comprehensive scoping review reveals that smartphones and mobile and ambient sensing systems have the potential to monitor users’ behaviors and daily activities to infer loneliness and social isolation, and it is likely that research interest in this field will grow in the future. However, most existing methods have shortcomings, particularly in privacy preservation and validation across diverse populations, which need to be rigorously addressed in future research. Finally, it is worth noting that researchers need to investigate what motivates people to use such tracking mechanisms and what inspires their trust and long-term adherence if they are to be adopted and implemented within wider populations.

## Appendix

Multimedia Appendix 1 The characteristics of the included studies.

## Abbreviations

PRISMA: Preferred Reporting Items for Systematic Reviews and Meta-Analyses
PRISMA-ScR: Preferred Reporting Items for Systematic Reviews and Meta-Analyses extension for Scoping Reviews
UCLA: University of California, Los Angeles
