# Supplementary material for: Loneliness and Social Isolation Detection Using Passive Sensing Techniques: Scoping Review
Source: JMIR Mhealth Uhealth. 2022 Apr 12;10(4):e34638. doi: 10.2196/34638 (PMC9044142; doi:10.2196/34638)
Supplement: Multimedia Appendix 1 [file mhealth_v10i4e34638_app1.docx]

**Multimedia Appendix 1. Table-2** The characteristics of the included studies.

| **Study**  **Authors** | **Year** | **Country** | **Population** | **Technology** | **Data Streams** | **Assessment** | **Indicators** | **Study**  **Design** |
| --- | --- | --- | --- | --- | --- | --- | --- | --- |
| [77]Petersen,  Johanna and  Austin | 2013 | USA | Older Adults, Data  collected through  homes of 150  independently living  seniors | Contact  Sensors | Sensors | UCLA | Outdoor Visit  Pattern | Observational |
| [58]Wang, Rui  and Chen | 2014 | USA | College Students, 48  Students | Smartphone  app | Accelerometer, light  sensor, microphone,  GPS, Bluetooth  encounters | MobileEMAs + 4 other  surveys including UCLA  pre/post study | Activity, sleep,  sociability,  conversation | Longitudinal |
| [63]Yow, W  Quin and Li | 2014 | Singapore | College Students, 35  First year College  Students | Smartphone  Sensors | GPS, Bluetooth scans,  Calls and SMS logs,  audio recordings | UCLA, Friendship Tie  Strength Questionnaire,  Classroom community scale,  Student adaption to college  questionnaire | Social Interaction,  Visited Locations,  Calls and SMS  Pattern | Longitudinal |
| [70]Walsh, Lorcan  and Kealy | 2014 | Ireland | Older Adults, 13 adults between 60 and 88 years old | Ambient  Sensors | Sensors | Anxiety(HADS), Sleep  quality (PSQI), Depression  (CES-D), Loneliness (De  Jong Giervald), Cognition  (MOCA), Quality of Life  (SF-36), Independent living  skills (IADL) | Sleep, Activity,  Movement | Observational |
| [75]Sanchez,  Wendy and  Martinez | 2015 | Mexico | Older Adults, 12  Participants | Smartphone  App | Call logs, GPS | ESTE-R Scale | Number of outgoing/  incoming  calls, number of  outings, average  time spent  inside/outside  home | Cross-  Sectional |
| [66]Ben-Zeev,  Dror and Scherer | 2015 | USA | Young Population, 47  Participants aged  between 19 to 30 years | Smartphone  App | Microphone, GPS, WIFI  Scanner, Accelerometer | UCLA |  | Longitudinal |
| [76]Lyons, Bayard E and Austin | 2015 | USA | Older Adults, 100s of  participants who were  70 years or older | In-home  Ambient  Sensors | Sensors | UCLA | Time-Spent Out  Of Home | Observational |
| [73]Austin, Johanna and  Dodge | 2016 | USA | Older Adults, 16 Participants | Wireless  Movement  detectors  and contact  sensors | Ambient Sensors,  Computer Usage Logs | UCLA | Hours Spent  Outside Home,  Number Of Incoming/  Outgoing  Calls and SMS,  Walking speed,  Mobility | Longitudinal |
| [65]Li,  Zhongqiu  and Shi | 2016 | China | Not Specified,  48 Students | Smartphone | App Usage, Call/SMS  Logs, GPS, Bluetooth | UCLA,PSS,PHQ-9 | Types of apps  being used,  Time spent on  phone usage | Longitudinal |
| [74]VandeWeerd,  C and Agu | 2016 | USA | Older Adults | Ambient sensors | Sensors, Bi-Weekly  Assessment (BWA)  Meetings | Time Spent Inside/Outside  Home |  | Observational |
| [64]Pulekar,  Gauri and Agu | 2016 | Libya | Any Age group,  9 Students | Smartphone  App | Call Logs, SMS logs,  Browsing Data, Emails,  And Social Media Usage | UCLA and Personality Survey  Questionnaire | No. and pattern  of SMS/calls  and browser  search activities | Longitudinal |
| [83]Gao, Yusong  and Li | 2016 | China | Not Specified,  146 Participants | Smartphone  App | Call/SMS Logs, Contact  Data, GPS, Screen  On/O | Mobile app based questionnaire  for getting basic info, IAS scale  and UCLA scale | Number Of  Outgoing  Calls/SMS | Longitudinal |
| [77]Petersen,  Johanna  and  Thielke | 2016 | USA | Older Adults, 26  Participants | Landline phone  as a monitoring  device | UCLA and weekly  health forms | Mobility, Voice, Activity |  | Observational |
| [80]Servia-  Rodrguez,  Sandra  and  Rachuri | 2017 | UK | Not Specified,  18K App Users | Smartphone  App | Accelerometer, GPS,  Microphone, SMS/Call  Logs | Two surveys per day related to  user's activities, health, personality, location and mood | Sleep,  Sociability,  Conversation | Cross-Sectional |
| [78]Huynh,  Sinh  and Tan | 2017 | Singapore | Older Adults, 50  Participants | Ambient Sensors | Sensors | Loneliness scale developed by  Gierveld and Geriatric  depression scale survey (GDS) | Less activity,  less out going  from home | Longitudinal |
| [69]Hu,  Rui and  Pham | 2017 | Switzerland | Older Adults, 5 Participants | Ambient Sensors  and FitBit | Sensors and Wearable | Caregivers intervention logs  including date, time and context  of visit are collected on regular  basis |  | Longitudinal |
| [72]Martinez,  Alicia  and  Ortiz | 2017 | Mexico | Older Adults, 7  Participants | Smartphone app  and beacons as  sensors | Calls and SMS Logs,  Beacons | Lubben Social Network Scale | Communication  Patterns,  Average time  spent in  different areas of  home | Cross-Sectional |
| [81]Gu,  Fei and  Niu | 2017 | China | Not Specified,  30 people in 6  families with  children and  elders | Apple Watch  and Smartphone  Sensors | Body sensing data and  environmental sensing  using microphone,  Accelerometer,  Magnetometer,  Gyroscope | A 5 items questionnaire to get  data about user's personal health  routine and family interaction  pattern | Hear Rate,  Strides, Motions | Longitudinal |
| [68]Goonawardene,  Nadee and Toh | 2017 | Singapore | Adults, 50  Participants | PIR Sensors | Sensors | Home Visits, Survey about  physical and mental wellbeing | Sleep, Going  Out, Activity in home | Observational |
| [61]Quiroz,  Juan Carlos  and  Geangu | 2018 | USA | Young  Population, 50  Participants | Wearable  Smartwatch | Heart rate and  Accelerometer data | Positive affect negative affect scores (PANAS) | Heart Rate,  Walking  Behaviour | Observational |
| [59]Doryab,  Afsaneh  and Villalba | 2019 | USA | College  Students, 160  Participants | Smartphones | Bluetooth Addresses,  WIFI, Location, Phone  Usage(lock/unlock  status), Calls and SMS  Logs | UCLA | Social  Interaction,  Sleep, Activity | Longitudinal |
| [62]Poudyal,  Anubhuti  and van  Heerden | 2019 | USA | Young Mothers,  40 Participants | Smartphone  App and  Beacons | Accelerometer, GPS,  Beacons, Audio  Recordings | PHQ-9 tool to measure  depression severity, BDI,  WHODAS, HOME | Physical  Activity,  Locations for  mood enhancing  activities, Social  Interaction,  Time spending  with child | Cross-Sectional |
| [82]Costa,  Angelo  and Rincon | 2019 | Portugal | Not Specified,  20 subjects with  a database of  1182 images | Wrist Bands | Galvanic Skin Response,  Photoplethysmogram,  Accelerometer,  Gyroscope | Not Used | Negative  Emotions | Observational |
| [60]Jacobson,  Nicholas  C and  Summers | 2020 | USA | Young  Population, 72  Students | Smartphone  App | Accelerometer, Calls and  SMS Logs | SIAS, PANAS, DASS-21 |  | Longitudinal |
| [84]Fulford,  Daniel  and Mote | 2020 | USA | 35 Participants | Smartphone  App | GPS and Ambient Audio | UCLA | Mobility, Voice  Activity | Cross-Sectional |
| [67]Wu,  Congyu  and  Barczyk | 2021 | USA | Young  Population, 129  Students | Smartphone  App | GPS and Bluetooth | Ecological momentary assessment  surveys (EMA) |  | Longitudinal |
| [85]Wetzel  et al. | 2021 | Germany | Adults (18-78  year old), 364  participants | Smartphone  communication  apps | App usage, phone and  video calls, SMS | Loneliness Scale-SOEP [LS-S],  World Health Organization  Quality of Life-Instrument  (WHOQOL-Bref) |  | Cross-sectional  / longitudinal |
| [92]Bellini  et al. | 2021 | Italy | Assisted care  home for people  with  Alzheimer's  disease | Bluetooth  bracelets | Localization data  captured through  antennas | Localization data to form the  social pro le of residents and  their social contacts | Popularity of  places, Strength  of relationship | Observational |
| [79] Leese, Mira I | 2021 | USA | 59 Older Adults | Monitoring software installed on computer, a passive driving monitoring sensor installed on vehicle | Monitoring software | Survey Questionnaires | Computer use, driving trips | Longitudinal |
